# Computational Analysis of Molnupiravir

**DOI:** 10.3390/ijms23031508

**Published:** 2022-01-28

**Authors:** Artem V. Sharov, Tatyana M. Burkhanova, Tugba Taskın Tok, Maria G. Babashkina, Damir A. Safin

**Affiliations:** 1 «Advanced Materials for Industry and Biomedicine» Laboratory, Kurgan State University, Sovetskaya Str. 63/4, 640020 Kurgan, Russia; sharow84@gmail.com (A.V.S.); t.m.burkhanova@utmn.ru (T.M.B.); maria.babashkina@mail.ru (M.G.B.); 2 Center for Enterprise Relations, Ural Federal University Named after the First President of Russia B.N. Yeltsin, Mira Str. 19, 620002 Ekaterinburg, Russia; 3 Innovation Center for Chemical and Pharmaceutical Technologies, Ural Federal University Named after the First President of Russia B.N. Yeltsin, Mira Str. 19, 620002 Ekaterinburg, Russia; 4 Institute of Chemistry, University of Tyumen, Volodarskogo Str. 6, 625003 Tyumen, Russia; 5 Department of Chemistry, Faculty of Arts and Sciences, University of Gaziantep, Gaziantep 27310, Turkey; ttaskin@gantep.edu.tr or taskin.tugba@gmail.com; 6 Department of Bioinformatics and Computational Biology, Institute of Health Sciences, University of Gaziantep, Gaziantep 27310, Turkey

**Keywords:** COVID-19, SARS-CoV-2, molnupiravir, virus, computational study, DFT, molecular docking

## Abstract

In this work, we report in-depth computational studies of three plausible tautomeric forms, generated through the migration of two acidic protons of the *N*^4^-hydroxylcytosine fragment, of molnupiravir, which is emerging as an efficient drug to treat COVID-19. The DFT calculations were performed to verify the structure of these tautomers, as well as their electronic and optical properties. Molecular docking was applied to examine the influence of the structures of the keto-oxime, keto-hydroxylamine and hydroxyl-oxime tautomers on a series of the SARS-CoV-2 proteins. These tautomers exhibited the best affinity behavior (−9.90, −7.90, and −9.30 kcal/mol, respectively) towards RdRp-RTR and Nonstructural protein 3 (nsp3_range 207–379-MES).

## 1. Introduction

**Figure 1:**
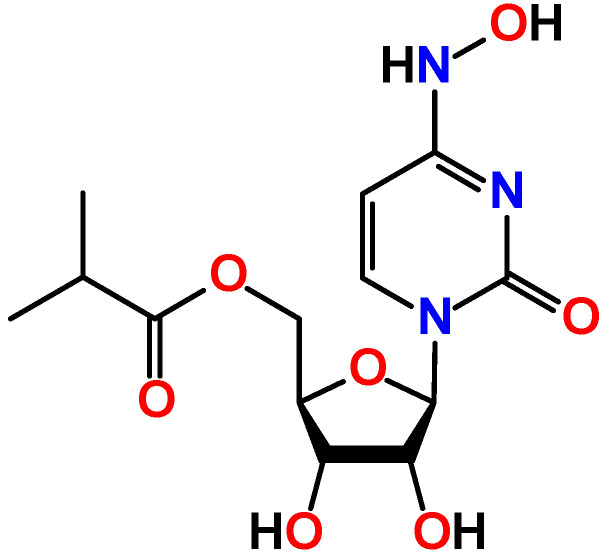
Diagram of molnupiravir.

Molnupiravir, which is known under the trademark Lagevrio, is a first oral antiviral for COVID-19 approved by Medicines and Healthcare products Regulatory Agency (MHRA) [1]. It is an *N*^4^-hydroxycytidine derivative, where the ribose residue is bonded to the isobutyric acid ester group (Figure 1). Molnupiravir was obtained at University of Emory (USA) but was refused due to mutagenicity. Later, rights on molnupiravir were purchased by a biotechnology company Ridgeback Biotherapeutics, which, in turn, partnered with Merck & Co, an American multinational pharmaceutical company, to perform clinical trials with molnupiravir in humans to treat COVID-19 [2]. Initially, molnupiravir was developed for the treatment of influenza [3], acting through integration into the replication process of the viral RNA. As a result, accumulation of a number of mutations does not allow the virus to maintain its own population [4,5].

Nowadays, coronavirus is one of the most discussed and actively investigated viruses. To be said, coronaviruses are a large family of viruses, which may cause illness in animals or humans. In humans, several coronaviruses are known to cause respiratory infections ranging from the common cold to more severe diseases such as Middle East Respiratory Syndrome (MERS) and Severe Acute Respiratory Syndrome (SARS). The most recently discovered coronavirus causes coronavirus disease COVID-19 [6]. Since the time when this disease was recognized, it has rapidly spread, and the World Health Organization (WHO) announced a pandemic in March 2020 [7]. As the causative agent of COVID-19, was found the betacoronavirus severe acute respiratory syndrome coronavirus 2 (SARS-CoV-2).

To date, the beginning of 2022, about 310 million infections were confirmed with about 5.5 million deaths [8]. A steady upward trend in this disease has been observed. Unfortunately, the situation with COVID-19 remains very complicated due new strains, of which variants of concern are alpha, beta, gamma, delta and omicron. The latter strain was first discovered in November 2021.

The dire need to search for antiviral agents to combat COVID-19 has led to the emergence of studies on the effectiveness of molnupiravir against SARS-CoV-2 [9,10,11,12,13,14,15,16]. As of October 2021, it was established that oral administration of molnupiravir reduces the risk of severe disease by about 50% in comparison to placebo in patients with mild to moderate disease. Furthermore, molnupiravir was found to be more efficient in comparison to other drugs against COVID-19 [17,18]. In addition, molnupiravir was also established to be effective against the omicron strain, since it interferes with how the virus replicates, a process that isn’t altered across variants [19,20].

All this dictates that molnupiravir is currently in the limelight of research and under an ever-growing interest. Thus, deeper properties of molnupiravir are revealed as a more powerful weapon against viruses, including COVID-19.

With all this in mind, as well as in continuation of our ongoing interest in in silico studies of bioactive compounds [21,22,23,24] we have directed our attention to molnupiravir. Theoretical calculations based on density functional theory (DFT) were performed to examine electronic and optical properties of its three tautomers. The global chemical reactivity descriptors were estimated from the energy of the HOMO and LUMO orbitals to examine the relative reactivity of the molecules. Using an in silico molecular docking method, we have explored the binding modes and interactions of each tautomer with binding sites of a series nonstructural proteins and the structural protein (Spike protein, RBD) of the SARS-CoV-2 as targets.

## 2. Results and Discussion

**Figure 2:**
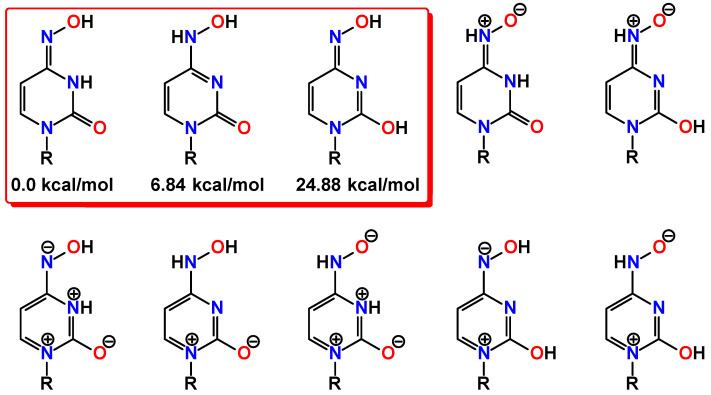
Diagrams of the plausible tautomers of the *N*^4^-hydroxylcytosine fragment in the molecule of molnupiravir (R = residue of the molecule of molnupiravir).

Molnupiravir can conventionally be considered as a molecule constructed from the two main structural fragments, namely the substituted ribose and *N*^4^-hydroxylcytosine (Figure 1). Due to the hydroxylamine group, the latter fragment can, in general, exhibit two tautomeric forms of either the hydroxylamine or oxime structure, of which the latter can further generate the amide-iminol tautomerism (Figure 2). Furthermore, two nitrone forms can also be highlighted as plausible tautomeric forms of molnupiravir also dictated by the amide-iminol transformation (Figure 2). Finally, a series of ionic aromatic forms are further tautomers of molnupiravir (Figure 2). Thus, two acidic protons of the *N*^4^-hydroxylcytosine fragment are of great importance and play a pivotal role in a rich library of plausible tautomeric forms of molnupiravir. Notably, to the best of our knowledge, the crystal structure of molnupiravir has not been reported so far. This can also be explained by intertautomer transformation in solutions.

Among the variety of tautomers of molnupiravir, herein, we have directed our attention to three forms without any charged centers, namely keto-oxime, keto-hydroxylamine, and hydroxyl-oxime (Figure 2). We have applied the DFT calculations to shed light on fine features of these tautomers. Each structure was first optimized in gas phase at 298.15 K, and the energies and thermodynamic parameters are given in Table 1.

It was established that the keto-oxime tautomer is the most energetically stable, followed by the keto-hydroxylamine tautomer, which is about 7 kcal/mol less favorable, while the hydroxyl-oxime tautomer is completely unfavorable (Figure 2). The calculated bond lengths between the non-hydrogen atoms are gathered in Table 2. Notably, all tautomers exhibit very similar bond lengths within the substituted ribose fragment, while differ in their *N*^4^-hydroxylcytosine parts (Table 2).

**Figure 3:**
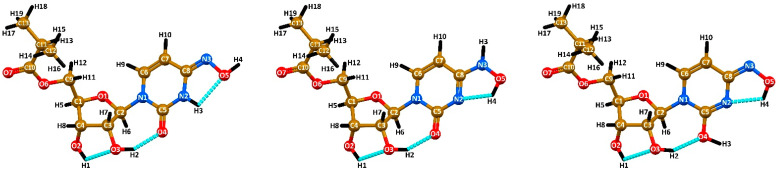
Optimized structures of the keto-oxime (**left**), keto-hydroxylamine (**middle**), and hydroxyl-oxime (**right**) tautomers of molnupiravir, obtained by using the B3LYP/6-311++G(d,p) method. Cyan dashed line = O–H∙∙∙O, O–H∙∙∙N and N–H∙∙∙O hydrogen bonds.

Each tautomeric form is stabilized by three hydrogen bonds. Particularly, the same hydrogen bond O–H∙∙∙O is formed in the tautomers between one of the hydroxyl hydrogen atoms and the next hydroxyl oxygen atom within the ribose cycle (Figure 3, Table 3). The second O–H∙∙∙O hydrogen bond is formed between the other hydroxyl hydrogen atom of the ribose residue and either with the carbonyl oxygen atom, in the keto-oxime and keto-hydroxylamine tautomers, or the third hydroxyl oxygen atom, in the hydroxyl-oxime tautomer, respectively (Figure 3, Table 3). Finally, the third N–H∙∙∙O or O–H∙∙∙N hydrogen bond is formed within the *N*^4^-hydroxylcytosine fragment (Figure 3, Table 3). As a result of these non-covalent interactions, each tautomer exhibits two five-membered and one seven-membered hydrogen bonded rings.

**Figure 4:**
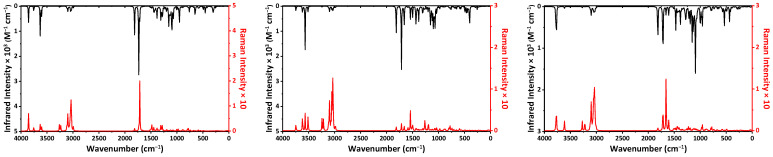
The calculated IR (black) and Raman (red) spectra of the keto-oxime (**left**), keto-hydroxylamine (**middle**), and hydroxyl-oxime (**right**) tautomers of molnupiravir, obtained by using the DFT/B3LYP/6-311++G(d,p) method.

The molecules of the discussed tautomeric forms of molnupiravir contain 42 atoms and, thus, have 120 normal modes (Table 4). All the frequencies were found to be positive, indicating local energy minima for the optimized structure. In general, both the IR and Raman spectra of each tautomer are informative for the C=O, OH, and NH groups (Figure 4). The most intense band in the calculated IR spectra of the keto-oxime and keto-hydroxylamine tautomers is observed at 1733 and 1715 cm^−1^, respectively (Figure 4, Table 4). In both spectra this band is associated with stretching of the carbonyl group of the cyclic fragment, bending of one of the ribose hydroxyl group together with bending of the amine group in the former spectrum or bending of the hydroxylamine OH group, and one of the CH groups of the dinitrogen containing cycle, respectively (Table 4). In the IR spectrum of the hydroxyl-oxime tautomer the most intense band is observed at 1092 cm^−1^ and is due to stretching of one of the ribose CO groups, bending of both CH groups of the dinitrogen containing cycle and rocking of the CH_2_ fragment.

According to the DFT calculations, the energies of the HOMO and LUMO are −6.36339 ÷ −5.84691 and −1.89092 ÷ −1.26370 eV, respectively, with the lowest and highest values corresponding to the keto-hydroxylamine and hydroxyl-oxime tautomers (Table 5). The corresponding energy gap varies from 4.58321 to 5.02322 eV (Table 5).

**Figure 5:**
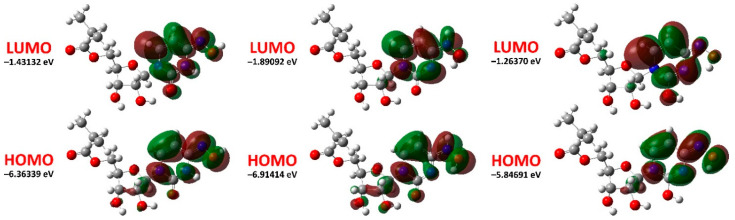
Energy levels and views on the electronic isosurfaces of the HOMO and LUMO of the optimized structures of the keto-oxime (**left**), keto-hydroxylamine (**middle**), and hydroxyl-oxime (**right**) tautomers of molnupiravir, obtained by using the B3LYP/6-311++G(d,p) method.

We have also visualized HOMO and LUMO for the discussed tautomers. It was found that both orbitals are mainly delocalized over the substituted dinitrogen fragment with some contribution from the ribose fragment for HOMO (Figure 5).

**Figure 6:**
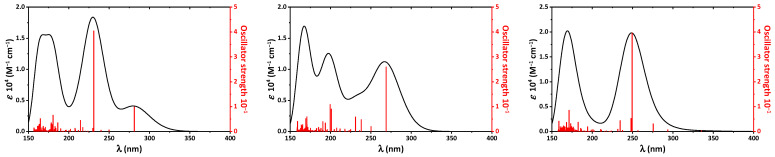
The calculated UV-vis spectra of the keto-oxime (**left**), keto-hydroxylamine (**middle**), and hydroxyl-oxime (**right**) tautomers of molnupiravir, obtained by using the TD-DFT/B3LYP/6-311++G(d,p) method.

The calculated UV-vis spectra of all tautomers exhibit bands exclusively in the UV region. Particularly, the spectrum of the keto-oxime tautomer contains bands at 168, 175, 230, and 280 nm (Figure 6), which mainly correspond to the transitions at 164.8, 178.3, 180.6, 186.4, 231.0, and 281.3 nm, respectively (Table 6). The latter two low-energy transitions are due to HOMO → LUMO and HOMO → LUMO+2 (Table 6). The spectrum of the keto-hydroxylamine tautomer exhibits bands centered at 168 (transitions at 159.1, 169.3, and 170.7 nm), 198 (transitions at 190.6, 199.6, and 201.1 nm), and 267 (transition at 267.0 nm) nm accompanied with a shoulder at about 235 (transitions at 231.0 and 238.0 nm) nm (Figure 6). The latter two low-energy transitions are mainly due to HOMO → LUMO, HOMO → LUMO+1 and HOMO → LUMO+2 (Table 6). The calculated absorption spectrum of the hydroxyl-oxime tautomer exhibits only two clearly distinguished bands at 169 (transitions at 158.7, 171.3, 173.4, 182.4, and 182.6 nm) and 248 (transitions at 234.6, 248.0, 249.6, and 275.5 nm) nm (Figure 6). The low-energy band is assigned to HOMO → LUMO+1÷7 (Table 6).

The ionization potential (*I*) and the electron affinity (*A*) value of the molecule, determined as *I* = −*E*_HOMO_ and *A* = −*E*_LUMO_ (Table 5) [25], are large indicating that the reported tautomers exhibit low electron donating and high electron accepting properties. Notably, the highest and lowest values of *I* and *A* were found for the keto-hydroxylamine and hydroxyl-oxime tautomers, respectively (Table 5).

We have further established values of the so-called global chemical reactivity descriptors. Chemical potential (*μ*) for the discussed tautomers varies from −4.40253 to −3.55531 eV, indicating electron accepting ability and the low donating ability, which is supported by the corresponding high value of electronegativity, *χ* (Table 5). The electrophilicity index (*ω*), which is denoted as the energy of stabilization to accept electrons [25], is 2.75794–3.85854 eV, indicating the pronounced electrophilic nature of the tautomers. Finally, the reported tautomers of molnupiravir can accept about 1.55–1.75 electrons as evidenced from the corresponding ΔN_max_ values (Table 5).

**Figure 7:**
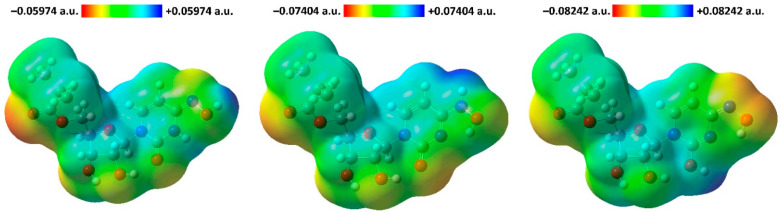
View of the molecular electrostatic potential surfaces of the optimized structures of the keto-oxime (**left**), keto-hydroxylamine (**middle**), and hydroxyl-oxime (**right**) tautomers of molnupiravir, obtained by using the B3LYP/6-311++G(d,p) method.

The electrophilic and nucleophilic sites in the discussed tautomers of molnupiravir were examined using the molecular electrostatic potential (MEP) analysis. The red and blue colours of the MEP surface correspond to electron-rich (nucleophilic) and electron-deficient (electrophilic) regions, respectively. On the MEP surface of the keto-oxime and keto-hydroxylamine tautomers the most pronounced nucleophilic centers are located on the carbonyl oxygen atom of the ester fragment followed by the carbonyl oxygen atom of the dinitrogen cycle and hydroxyl oxygen atoms (Figure 7). As the most electrophilic region in the former tautomer the hydroxyl hydrogen atom of the oxime fragment can be highlighted, while in the latter tautomer, the amine hydrogen atom is the most electrophilic site (Figure 7). Interestingly, in the hydroxyl-oxime tautomer the most pronounced nucleophilic centers are located on the nitrogen and oxygen atoms of the oxime fragments, followed by the carbonyl oxygen atom of the ester fragment, while the most electrophilic site was found on the hydrogen atom of the hydroxyl group attached to the dinitrogen cycle (Figure 7).

The calculated ^1^H NMR spectra of the reported tautomers of molnupiravir each contain a set of signals, characteristic for protons of a certain nature. Particularly, the CH_3_ and CH protons of the isopropyl group and CH_2_ protons are observed at 0.71–1.62, 2.38–2.46, and 3.53–4.05 ppm, respectively (Table 7). The signals for the CH protons of the ribose and dinitrogen cycles are found at 3.77–5.59 and 5.24–7.80 ppm, respectively (Table 7). Furthermore, while the signals for the H1 hydroxyl protons are shown almost in the same region at 2.33–2.90 ppm, the signals for the other hydroxyl protons, together with the signals for the NH protons, vary from 2.87 to 7.88 ppm (Table 7). Notably, the calculated ^1^H NMR spectra of both the keto-oxime and hydroxyl-oxime tautomers agree with the experimental one [26]. Since the experimental spectrum was recorded in methanol-*d*_4_, thus, vanishing plausible signals from the hydroxyl and amine hydrogens, it is impossible to clearly attribute the exact tautomer of the mentioned two. However, since the keto-oxime tautomer is much more energetically favorable in comparison to the hydroxyl-oxime tautomer, we can tentatively assign the experimental ^1^H NMR spectrum to the former tautomer.

To examine the potential nonlinear optical properties of three discussed tautomers of molnupiravir, parameters of the dipole moment (μ), polarizability (α), anisotropy of polarizability (Δα), and first-order hyperpolarizability (β) [27,28] were computed using the B3LYP/6-311++G(d,p) method (Table 8). The calculated dipole moment significantly increases from the hydroxyl-oxime through the keto-hydroxylamine to the keto-oxime tautomer. Such pronounced dipole moments for the latter two tautomers are due to the overall imbalance in the charge from one side of a molecule to the other side, which is also supported by the corresponding MEP surfaces (Figure 7). Thus, the presence of the keto-fragment formed within the dinitrogen cycle plays a pivotal role to increase the dipole moment of molnupiravir. Notably, an absolute value of the μ_y_ component exhibits the highest magnitude for the same two tautomers. Values for the calculated polarizability and first-order hyperpolarizability parameters for the three tautomers of molnupiravir are about 8.0 and 6.0–12.1 times higher in comparison to those of urea (Table 8), which is commonly used as a refernece for studying the nonlinear optical (NLO) properties of the molecular systems [29]. Thus, molnupiravir is of potential interest for future studies of its NLO properties.

Molnupiravir is known to be mutagenic [30]. Interestingly, using the OSIRIS Property Explorer software [31], we have established that while the keto-hydroxylamine tautomer is indeed mutagenic, its keto-oxime and hydroxyl-oxime derivatives do not possess mutagenic properties. Furthermore, the latter two tautomeric forms of molnupiravir exhibit more potent drug-likeness and drug-scores (−0.065 and 0.410 for keto-oxime, and −1.284 and 0.336 for hydroxyl-oxime, respectively) in comparison to those of the former tautomer (−2.529 and 0.178, respectively). Thus, the tautomeric form of molnupiravir is of importance in terms of drug safety.

We have further applied a molecular docking approach for all the three tautomers of molnupiravir against a series of SARS-CoV-2 proteins. The molecular docking aids in visualization and explication of the interaction between a small compound as ligand and biomolecule(s) as target(s) [32]. This application is one of the most broadly exerted technique to examine the structure-activity relationship and biological activity in the drug discovery [33]. Docking is the best option to diminish the time and cost of synthesis and to increase the influences of the medicines. In addition, it is considered as a current and advantageous method to have insight information of the possible binding site of the ligand in the protein [34].

In this study, molecular docking was employed to rationalize the three tautomers of molnupiravir in the SARS-CoV-2 targets. The target structures were primarily selected in accordance with the structural features of the virus [35,36] as well as based on biological mechanisms and functions that can be utilized to reduce, prevent, or treat the virus [37] (Table 9).

**Figure 8:**
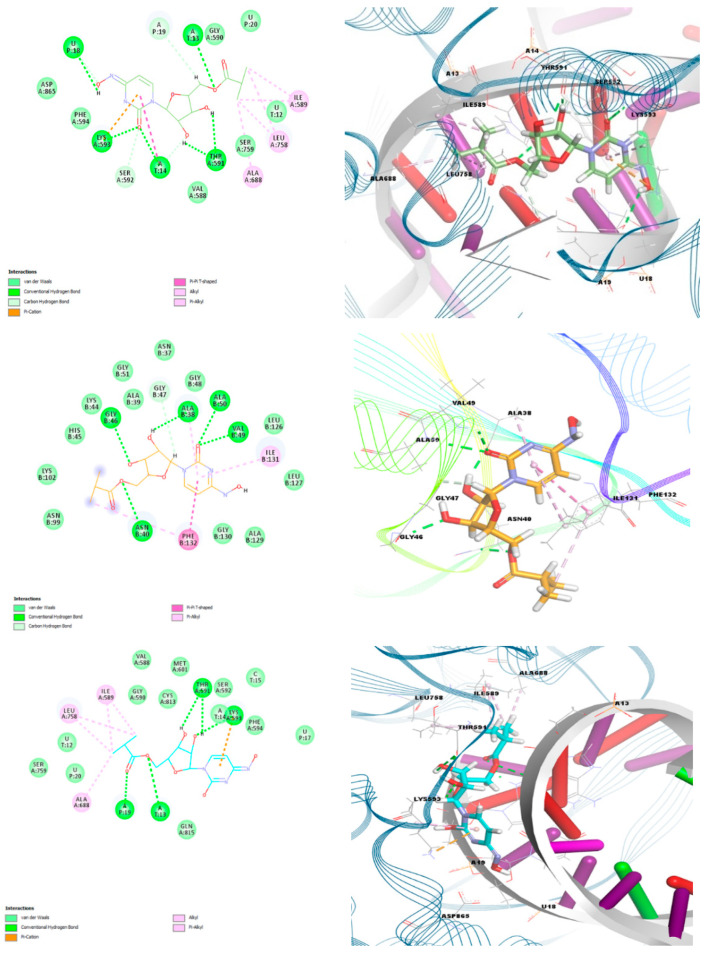
Two-dimensional (**left**) and 3D (**right**) views on the interaction of the keto-oxime (**top**), keto-hydroxylamine (**middle**), and hydroxyl-oxime (**bottom**) tautomers of molnupiravir with (from top to bottom) RdRp-RTR, Nonstructural protein 3 (Nsp3_range 207–379-MES) and RdRp-RTR.

According to the docking analyses results, both the keto-oxime and hydroxyl-oxime tautomers show the best binding affinity with the RdRp-RTR protein, while the keto-hydroxylamine tautomer is more efficient towards the nonstructural protein 3 (Nsp3_range 207–379-MES) (Figure 8, Table 9).

Complex of the keto-oxime tautomer with RdRp-RTP is described with the following interactions: eleven hydrogen bonds with LYS593, T:A13, T:A14, P:U18, THR591, SER592, and P:A19; one π-system∙∙∙cation interaction with LYS593; one T-shaped π∙∙∙π interaction with T:A14; five alkyl interactions with ALA688, ILE589, and LEU758; and one π-system∙∙∙alkyl interaction with LYS593 (Figure 8, Table S1 in the Supplementary Materials). The hydroxyl-oxime tautomer of molnupiravir forms a complex with the same protein through five hydrogen bonds with LYS593, P:A19, T:A13, and THR591; one π-system∙∙∙cation interaction with LYS593; five alkyl interactions with ALA688, ILE589, and LEU758; and one π-system∙∙∙alkyl interaction with LYS593 (Figure 8, Table S1 in the Supplementary Materials). Thus, these two tautomeric forms of monupiravir exhibit similar docking properties with the RdRp-RTP protein. For the keto-hydroxylamine tautomer of molnupiravir the most efficient interaction was found with the Nonstructural protein 3 (Nsp3_range 207–379-MES) through six hydrogen bonds with ASN40, GLY46, VAL49, ALA38, ALA50, and GLY47; one T-shaped π∙∙∙π interaction with PHE132; and three π-system∙∙∙alkyl interactions with PHE132, ALA38, and ILE131 (Figure 8, Table S1 in the Supplementary Materials).

Although none of the reported tautomers of molnupiravir showed superior binding scores with the main protease, Mpro (Table 9), this protein is a potential important drug target for coronavirus infections due to its essential role in processing the polyproteins that are translated from the viral RNA [38]. It was established that the keto-oxime tautomer of molnupiravir interacts with Mpro through seven hydrogen bonds formed with GLY143, SER144, CYS145, HIS163, LEU141, and GLN186; two alkyl interactions with MET165; and two π-system∙∙∙alkyl interactions with HIS41 and CYS145 (Table S2 in the Supplementary Materials). The keto-hydroxylamine forms complex with Mpro due to ten hydrogen bonds with GLY143, HIS163, GLU166, LEU141, SER144, MET165, and GLN198; two alkyl interactions with MET49 and MET165; and three π-system∙∙∙alkyl interactions with HIS41 and CYS145 (Table S2 in the Supplementary Materials). Finally, the hydroxyl-oxime tautomer of molnupiravir, which is the least efficiently bound to Mpro among the three reported tautomers (Table 9), interacts with the main protease via four hydrogen bonds with CYS145, LEU141, and PHE140; two alkyl interactions with MET49 and MET165; and three π-system∙∙∙alkyl interactions with HIS41 and CYS145 (Table S2 in the Supplementary Materials). Interestingly, one of the π-system∙∙∙alkyl interactions for all the tautomers with Mpro is formed by the π-system of the ligands (Figure S1 and Table S2 in the Supplementary Materials).

Besides the nonstructural proteins of SARS-CoV-2, spike protein, which is the structural protein, is of importance. The surface spike glycoprotein is consisting of two heterodimers S1 and S2. The receptor binding domain (RBD) is located on the head of S1 and binds the cellular receptor angiotensin-converting enzyme 2 (ACE2), initiating the membrane fusion of the virus and host cell. At this point, eight mutations (Y453F, L455F, F456L, A475V, A475S, T500S, N501Y, and Y505H) in the RBD and hACE2 interaction region (RBD/hACE2) were used to investigate the interaction mechanism of the reported tautomers of molnupiravir tautomers towards Spike protein, RBD as a target [39].

**Figure 9:**
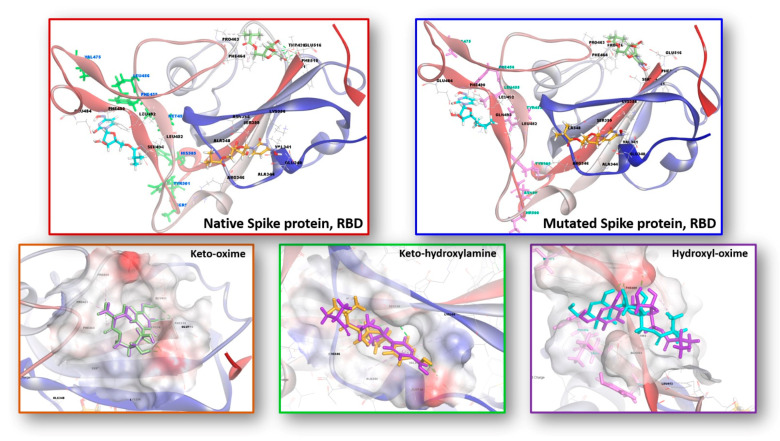
Interaction of the keto-oxime (green), keto-hydroxylamine (orange), and hydroxyl-oxime (cyan) tautomers of molnupiravir with the native (top **left**) and mutated (top **right**) Spike proteins, RBD. Behaviors of tautomers of molnupiravir towards native and mutated Spike proteins, RBD of SARS-CoV-2. The bottom row depicts the superimposed binding poses of the reported tautomers of molnupiravir with the native (purple) and mutated (green, orange and cyan) Spike proteins, RBD.

As a result of the calculations, while the binding affinity of the keto-hydroxylamine tautomer towards the mutated spike protein, RBD slightly decreased, the binding affinity of the keto-oxime and hydroxyl-oxime tautomers increased (Table 9). This is obviously explained by a different landscape of noncovalent interactions between the corresponding ligand and the target (Figure 9, Table S3 in the Supplementary Materials). As such, an interesting finding can be highlighted for the interaction of the hydroxyl-oxime tautomer of molnupiravir with the spike protein, RBD. Particularly, this tautomer interacts with the native spike protein, RBD exclusively through a set of hydrogen bonds and alkyl interactions, while π-system∙∙∙alkyl interactions were revealed for binding of the native spike protein, RBD with all the tautomers of molnupiravir, and for binding of the mutated spike protein, RBD with the keto-oxime and keto-hydroxylamine tautomers of molupiravir (Table S3 in the Supplementary Materials).

## 3. Methods

### 3.1. DFT Calculations

The ground state geometries of the keto-oxime, keto-hydroxylamine and hydroxyl-oxime tautomers of molnupiravir were fully optimized without symmetry restrictions. The calculations were performed by means of the GaussView 6.0 molecular visualization program [40] and Gaussian 09, Revision D.01 program package [41] using the density functional theory (DFT) method with Becke-3-parametr-Lee-Yang-Parr (B3LYP) hybrid functional [42,43] and 6-311++G(d,p) [42,44] basis set. The vibration frequencies, as well as nonlinear optical properties (polarizability and first-order hyper-polarizability), were calculated for the optimized structures in gas phase and no imaginary frequencies were obtained. The electronic isosurfaces of the HOMO and LUMO orbitals and MEP surfaces were generated from the fully optimized ground state geometries obtained by using the B3LYP/6-311++G(d,p) method. The absorption and ^1^H NMR spectra of the fully optimized ground state geometries of the discussed tautomers were simulated at the TD-DFT/B3LYP/6-311++G(d,p) and GIAO/B3LYP/6-311++G(2d,p) levels, respectively.

### 3.2. Molecular Docking

Molecular docking simulations of the keto-oxime, keto-hydroxylamine, and hydroxyl-oxime tautomers of molnupiravir with a series of the SARS-CoV-2 proteins were carried out with AutoDock Vina [45,46]. The targeted protein structures were acquired via the RCSB PDB database [47], and were pre-treated before the docking, including water removing and inserting hydrogen atoms and missing residues and charges [23]. The ligands were optimized using the DFT/B3LYP/6-311++G(d,p) [42,44] basis set. Autodock Tools 1.5.7 was utilized to define the grid box with the dimensions of 30 × 30 × 30 size. During the docking procedure, 200 conformations for each ligand were left flexible, while the protein was held rigid. The lowest binding energy conformers and two dimensional (2D) interactions were filtered from 10 top ranked poses. Discovery Studio 3.5 [48] was utilized for visualization of the docked conformations and 3D target-ligand interactions.

## 4. Conclusions

In summary, we report detailed computational analysis of molnupiravir, which is emerging as an efficient drug to treat COVID-19. We have focused on three plausible tautomeric forms of molnupiravir, formed due to two acidic protons of the *N*^4^-hydroxylcytosine fragment, namely keto-oxime, keto-hydroxylamine and hydroxyl-oxime. According to the DFT/B3LYP/6-311++G(d,p) calculation results, it was established that the keto-oxime tautomer is the most energetically stable, followed by the keto-hydroxylamine tautomer, which is about 7 kcal/mol less favorable, while the hydroxyl-oxime tautomer is completely unfavorable.

We have also calculated IR, Raman, ^1^H NMR and absorption spectra, which were fully described and identified. We have also established values of the global chemical reactivity descriptors, which revealed that the discussed tautomers exhibit electron accepting ability and the low donating ability. Furthermore, values for the calculated polarizability and first-order hyperpolarizability parameters for tautomers are remarkably higher in comparison to those of urea, which is commonly used as a reference for studying the nonlinear optical (NLO) properties of the molecular systems. Thus, molnupiravir is of potential interest for future studies of its NLO properties.

In silico molecular docking was applied to probe interactions of the three tautomers of molnupiravir with a series of the SARS-CoV-2 proteins. It was established that both the keto-oxime and hydroxyl-oxime tautomers show the best binding affinity with the RdRp-RTR protein, while the keto-hydroxylamine tautomer is more efficient towards the Nonstructural protein 3 (Nsp3_range 207–379-MES). It was also established that the binding affinity of the keto-hydroxylamine tautomer towards the mutated Spike protein, RBD slightly decreased, while the binding affinity of the keto-oxime and hydroxyl-oxime tautomers increased in comparison to the native Spike protein, RBD.

We hope that the results reported herein will be of value for future design of potential drugs as well as developing new efficient therapies against SARS-CoV-2.

## Figures and Tables

**Table 1:** Thermodynamic parameters of the optimized structures of the keto-oxime, keto-hydroxylamine, and hydroxyl-oxime tautomers of molnupiravir, obtained by using the DFT/B3LYP/6-311++G(d,p) method.

Thermodynamic Parameter	Keto-oxime	Keto-hydroxylamine	Hydroxyl-oxime
Self-consistent field energy (a.u.)	−1197.962	−1197.951	−1197.922
Total energy (thermal) (kcal mol^−1^)	225.112	224.768	224.572
Electronic energy (thermal) (kcal mol^−1^)	0.000	0.000	0.000
Translational energy (thermal) (kcal mol^−1^)	0.889	0.889	0.889
Rotational energy (thermal) (kcal mol^−1^)	0.889	0.889	0.889
Vibrational energy (thermal) (kcal mol^−1^)	223.335	222.990	222.794
Total heat capacity (thermal) (cal mol^−1^ K^−1^)	85.340	84.962	86.088
Electronic heat capacity (thermal) (cal mol^−1^ K^−1^)	0.000	0.000	0.000
Translational heat capacity (thermal) (cal mol^−1^ K^−1^)	2.981	2.981	2.981
Rotational heat capacity (thermal) (cal mol^−1^ K^−1^)	2.981	2.981	2.981
Vibrational heat capacity (thermal) (cal mol^−1^ K^−1^)	79.379	79.001	80.127
Total entropy (thermal) (cal mol^−1^ K^−1^)	164.370	163.370	165.868
Electronic entropy (thermal) (cal mol^−1^ K^−1^)	0.000	0.000	0.000
Translational entropy (thermal) (cal mol^−1^ K^−1^)	43.269	43.269	43.269
Rotational entropy (thermal) (cal mol^−1^ K^−1^)	35.425	35.425	35.428
Vibrational entropy (thermal) (cal mol^−1^ K^−1^)	85.676	84.677	87.170
Zero-point vibrational energy (thermal) (kcal mol^−1^)	210.837	210.592	210.174
Rotational constants (GHz)			
*A*	0.38535	0.39715	0.41586
*B*	0.12511	0.12266	0.11980
*C*	0.10302	0.10192	0.09932

**Table 2:** Selected bond lengths (Å) in the optimized structures of the keto-oxime, keto-hydroxylamine, and hydroxyl-oxime tautomers of molnupiravir, obtained by using the DFT/B3LYP/6-311++G(d,p) method.

Bond	Keto-oxime	Keto-hydroxylamine	Hydroxyl-oxime
C1–C4	1.525	1.526	1.529
C1–C9	1.528	1.527	1.527
C2–C3	1.551	1.551	1.546
C3–C4	1.539	1.540	1.538
C6–C7	1.344	1.358	1.341
C7–C8	1.443	1.426	1.451
C10–C11	1.528	1.528	1.527
C11–C12	1.544	1.544	1.544
C11–C13	1.534	1.534	1.534
C1–O1	1.451	1.452	1.452
C2–O1	1.409	1.411	1.409
C3–O3	1.405	1.403	1.413
C4–O2	1.416	1.415	1.416
C5–O4	1.228	1.229	1.359
C9–O6	1.432	1.431	1.430
C10–O6	1.369	1.369	1.371
C10–O7	1.200	1.200	1.200
C5–N1	1.384	1.422	1.365
C5–N2	1.373	1.358	1.281
C6–N1	1.395	1.363	1.409
C8–N2	1.396	1.323	1.407
C8–N3	1.289	1.359	1.291
N3–O5	1.421	1.398	1.389

**Table 3:** Hydrogen bond lengths (Å) and angles (°) in the optimized structures of the keto-oxime, keto-hydroxylamine and hydroxyl-oxime tautomers of molnupiravir, obtained by using the DFT/B3LYP/6-311++G(d,p) method.

Tautomer	D–X∙∙∙A	*d*(D–X)	*d*(X∙∙∙A)	*d*(D∙∙∙A)	∠(DXA)
keto-oxime	O2–H1∙∙∙O3	0.968	2.095	2.648	114.52
	O3–H2∙∙∙O4	0.974	1.843	2.718	147.92
	N2–H3∙∙∙O5	1.012	2.163	2.537	99.62
keto-hydroxylamine	O2–H1∙∙∙O3	0.967	2.165	2.676	111.59
	O3–H2∙∙∙O4	0.966	2.064	2.917	146.30
	O5–H4∙∙∙N2	0.975	2.032	2.622	116.97
hydroxyl-oxime	O2–H1∙∙∙O3	0.967	2.165	2.676	111.59
	O3–H2∙∙∙O4	0.966	2.064	2.917	146.30
	O5–H4∙∙∙N2	0.975	2.032	2.622	116.97

**Table 4:** Values of the selected vibrations in the calculated IR and Raman spectra (Figure 4) for the optimized structures of the keto-oxime, keto-hydroxylamine, and hydroxyl-oxime tautomers of molnupiravir, obtained by using the DFT/B3LYP/6-311++G(d,p) method.

Molecular Vibration ^1^	Frequency (cm^−1^)	IR Intensity (KM∙mol^−1^)	Raman Activity (Å^4^∙amu^−1^)	Force Constant, *k* (mDyne A^−1^)
keto-oxime				
*ν*O5–H4	3856	189.04	220.68	9.3559
*ν*O2–H3	3754	73.43	60.22	8.8462
*ν*O3–H2	3629	417.06	96.23	8.2735
*ν*NH	3605	109.93	48.16	8.2416
*ν*_s_(C6–H9 + C7–H10)	3261	12.72	74.49	6.8628
*ν*_as_(C6–H9 + C7–H10)	3237	6.62	66.59	6.7326
*ν*C13–H14	3120	20.70	48.34	6.3159
*ν*_as_H14–C12–H15	3106	15.07	37.91	6.2675
*ν*C1–H5	3102	21.15	84.32	6.1803
*ν*C13–H18	3099	25.87	71.32	6.2376
*ν*_as_H15–C12–H16	3095	29.03	43.98	6.2194
*ν*_as_H11–C9–H12 + *ν*C1–H5	3077	13.81	48.81	6.1665
*ν*C11–H13	3058	14.12	130.10	5.9645
*ν*C2–H6	3046	37.37	58.52	5.9362
*ν*C4–H8 + *ν*C2–H6	3043	22.81	157.47	5.9235
*ν*_s_(C13–H17 + C13–H18 + C13–H19)	3037	22.14	204.18	5.6303
*ν*_s_(C12–H14 + C12–H15 + C12–H16)	3031	25.20	121.28	5.6016
*ν*_s_(C9–H11 + C9–H12)	3027	19.80	43.60	5.7009
*ν*C3–H7	2994	23.23	49.13	5.7226
*ν*C10=O7	1814	367.75	20.51	22.8748
*ν*C5=O4 + *β*N2–H3 + *β*O3–H2	1733	836.72	22.71	11.9387
*ν*C8=N3 + *ν*C5=O4 + *ν*C6=C7 +*β*O5–H4 + *β*N2–H3 + *β*C6–H9 + *β*C7–H10	1714	140.42	371.53	13.9766
keto-hydroxylamine				
*ν*O2–H1	3745	76.60	59.88	8.8028
*ν*N3–H3	3619	108.95	137.56	8.3276
*ν*O3–H2	3569	526.21	126.65	8.0039
*ν*O5–H4	3512	85.58	129.66	7.7383
*ν*_s_(C6–H9 + C7–H10)	3244	9.42	77.27	6.7877
*ν*_as_(C6–H9 + C7–H10)	3216	0.59	82.40	6.6483
*ν*C13–H17 + *ν*C13–H18 + *ν*C13–H19	3120	19.99	48.02	6.3164
*ν*C12–H14 + *ν*C12–H15 + *ν*C12–H16	3106	15.40	37.56	6.2662
*ν*C1–H5	3101	20.69	86.39	6.1751
*ν*_as_H18–C13–H19)	3099	26.15	71.95	6.2365
*ν*_as_H15–C12–H16)	3095	28.63	44.13	6.2181
*ν*_as_H11–C9–H12 + *ν*C1–H5	3075	14.97	46.70	6.1604
*ν*C11–H13	3056	13.35	110.25	5.9559
*ν*C2–H6	3055	21.17	105.53	5.9716
*ν*C4–H8	3041	33.92	129.47	5.9150
*ν*_s_(C13–H17 + C13–H18 + C13–H19)	3037	22.96	204.79	5.6307
*ν*_s_(C12–H14 + C12–H15 + C12–H16)	3031	23.47	110.87	5.5975
*ν*_s_H11–C9–H12	3025	21.21	50.87	5.6943
*ν*C3–H7	2987	27.06	45.99	5.6959
*ν*C10=O7	1815	365.00	19.83	22.8983
*ν*C5=O4 + *β*O5–H4 + *β*O3–H2 + *β*C6–H9	1715	785.23	33.24	17.0337
*ν*C8=N3 + *ν*C5=O4 + *ν*C6=C7 +*β*C6–H9 + *β*C7–H10 + *β*C2–H6	1663	291.45	25.55	10.4411
*β*N3–H3 + *β*O5–H4	1591	20.24	20.25	2.7871
hydroxyl-oxime				
*ν*O2–H1 + *ν*O3–H2 + *ν*O4–H3	3782	94.84	36.15	8.9787
*ν*O2–H1 + *ν*O4–H3	3774	27.16	36.51	8.9377
*ν*O5–H4	3615	16.28	90.73	8.2026
*ν*C6–H9	3273	9.92	75.23	6.9078
*ν*C7–H10	3222	2.37	85.81	6.6768
*ν*C13–H17 + *ν*C13–H18 + *ν*C13–H19	3121	19.94	49.24	6.3195
*ν*C12–H14 + *ν*C12–H15 + *ν*C12–H16	3106	14.34	42.24	6.2714
*ν*_as_H18–C13–H19	3100	23.66	67.74	6.2426
*ν*C1–H5 + *ν*_as_H11–C9–H12	3099	22.56	82.80	6.1714
*ν*C12–H14 + *ν*C12–H15 + *ν*C12–H16	3095	30.72	47.70	6.2155
*ν*C1–H5 + *ν*_as_H11–C9–H12	3077	12.97	50.18	6.1565
*ν*C2–H6	3061	20.82	56.13	5.9950
*ν*C11–H13	3056	14.50	140.64	5.9601
*ν*C4–H8	3047	29.86	139.46	5.9408
*ν*(C13–H17 + C13–H18 + C13–H19)	3038	21.16	199.10	5.6333
*ν*(C12–H14 + C12–H15 + C12–H16)	3031	24.44	122.92	5.5991
*ν*_s_H11–C9–H12	3025	23.18	61.34	5.7005
*ν*C3–H7	3006	17.27	35.96	5.7667
*ν*C10=O7	1817	363.72	20.82	22.9946
*ν*C5=N2 + *ν*C6=C7 + *β*O4–H3 + *β*C6–H9 + *β*C7–H10	1718	556.37	146.50	12.9824
*ν*C8=N3 + *ν*C5=N2 + *ν*C6=C7 +*β*O4–H3 + *β*C7–H10	1658	71.05	288.48	13.6289
*ν*C8=N3 + *ν*C5=N2 + *ν*C6=C7 +*β*O4–H3 + *β*O5–H4 + *β*C6–H9 + *β*C7–H10	1609	85.65	63.89	13.3853

^1^ *ν*—stretching, *ν*_s_—symmetric stretching, *ν*_as_—antisymmetric stretching, *β*—bending.

**Table 5:** Frontier molecular HOMO and LUMO orbitals, gap value, and descriptors for the optimized structures of the keto-oxime, keto-hydroxylamine, and hydroxyl-oxime tautomers of molnupiravir, obtained by using the DFT/B3LYP/6-311++G(d,p) method.

Parameter	Keto-oxime	Keto-hydroxylamine	Hydroxyl-oxime
*E*_HOMO_ (eV)	−6.36339	−6.91414	−5.84691
*E*_LUMO_ (eV)	−1.43132	−1.89092	−1.26370
Δ*E*_LUMO−HOMO_ = *E*_LUMO_ − *E*_HOMO_ (eV)	4.93207	5.02322	4.58321
Ionization energy, *I* = −*E*_HOMO_ (eV)	6.36339	6.91414	5.84691
Electron affinity, *A* = −*E*_LUMO_ (eV)	1.43132	1.89092	1.26370
Electronegativity, *χ* = (*I* + *A*)/2 (eV)	3.89736	4.40253	3.55531
Chemical potential, *μ* = −*χ* (eV)	−3.89736	−4.40253	−3.55531
Global chemical hardness, *η* = (*I* − *A*)/2 (eV)	2.46604	2.51161	2.29161
Global chemical softness, *S* = 1/(2*η*) (eV^−1^)	0.20275	0.19908	0.21819
Global electrophilicity index, *ω* = *μ*^2^/(2*η*) (eV)	3.07972	3.85854	2.75794
Maximum additional electric charge, ΔN_max_ = −*μ*/*ƞ*	1.58041	1.75287	1.55145

**Table 6:** Values of the calculated UV-vis spectra (Figure 6) for the optimized structures of the keto-oxime, keto-hydroxylamine, and hydroxyl-oxime tautomers of molnupiravir, obtained by using the TD-DFT/B3LYP/6-311++G(d,p) method.

λ_max_ (nm)	Osc. Strength	Transition	λ_max_ (nm)	Osc. Strength	Transition
keto-oxime					
164.8	0.0514	HOMO−7 → LUMO+3 (32.5%)	186.4	0.0353	HOMO−8 → LUMO (16.5%)
		HOMO−6 → LUMO+5 (5.8%)			HOMO−2 → LUMO+2 (11.6%)
		HOMO−5 → LUMO+5 (7.2%)			HOMO−1 → LUMO+2 (47.8%)
178.3	0.0351	HOMO−3 → LUMO+2 (21.2%)	214.5	0.0445	HOMO−5 → LUMO (8.9%)
		HOMO → LUMO+15 (11.6%)			HOMO−2 → LUMO (13.4%)
		HOMO → LUMO+16 (27.3%)			HOMO−1 → LUMO (36.5%)
180.6	0.0655	HOMO−4 → LUMO+1 (8.0%)			HOMO → LUMO+6 (18.4%)
		HOMO−3 → LUMO+1 (14.4%)	231.0	0.4040	HOMO → LUMO (15.2%)
		HOMO−2 → LUMO+2 (43.7%)			HOMO → LUMO+2 (64.7%)
		HOMO−1 → LUMO+2 (9.5%)	281.3	0.0987	HOMO → LUMO (79.3%)
					HOMO → LUMO+2 (17.5%)
keto-hydroxylamine					
159.1	0.0406	HOMO−15 → LUMO (7.8%)	199.6	0.0340	HOMO−1 → LUMO+3 (29.8%)
		HOMO−13 → LUMO (9.0%)			HOMO−1 → LUMO+4 (28.8%)
		HOMO−9 → LUMO+1 (16.9%)			HOMO → LUMO+6 (13.5%)
		HOMO−9 → LUMO+2 (34.7%)		0.1085	HOMO−1 → LUMO+2 (23.1%)
169.3	0.0516	HOMO−10 → LUMO (19.8%)			HOMO → LUMO+5 (25.1%)
		HOMO−3 → LUMO+7 (9.2%)			HOMO → LUMO+6 (14.2%)
		HOMO−2 → LUMO+7 (10.6%)			HOMO → LUMO+7 (9.8%)
		HOMO−1 → LUMO+10 (7.3%)	201.1	0.0902	HOMO−1 → LUMO+2 (50.2%)
170.7	0.0589	HOMO−10 → LUMO (23.3%)			HOMO → LUMO+5 (20.6%)
		HOMO−2 → LUMO+7 (7.7%)	231.0	0.0581	HOMO → LUMO+2 (70.8%)
		HOMO−1 → LUMO+10 (12.0%)			HOMO → LUMO+4 (9.9%)
190.6	0.0396	HOMO−5 → LUMO+2 (8.0%)	238.0	0.0473	HOMO → LUMO+1 (73.5%)
		HOMO−3 → LUMO+2 (8.5%)			HOMO → LUMO+2 (9.9%)
		HOMO−2 → LUMO+1 (22.9%)	267.0	0.2593	HOMO → LUMO (85.7%)
		HOMO−2 → LUMO+2 (35.1%)			
hydroxyl-oxime					
158.7	0.0413	HOMO−9 → LUMO+2 (8.7%)	182.4	0.0330	HOMO−5 → LUMO (8.7%)
		HOMO → LUMO+32 (10.7%)			HOMO−4 → LUMO+1 (50.6%)
		HOMO → LUMO+34 (10.2%)			HOMO−3 → LUMO+2 (11.7%)
168.1	0.0366	HOMO−9 → LUMO (22.2%)	182.6	0.0374	HOMO−5 → LUMO (38.7%)
		HOMO−8 → LUMO+1 (7.5%)			HOMO−3 → LUMO+2 (15.6%)
		HOMO−1 → LUMO+7 (10.3%)			HOMO−2 → LUMO+3 (16.5%)
		HOMO → LUMO+30 (8.7%)	234.6	0.0434	HOMO → LUMO+6 (7.1%)
171.3	0.0855	HOMO−9 → LUMO (15.4%)			HOMO → LUMO+7 (77.8%)
		HOMO−8 → LUMO+1 (16.6%)	248.0	0.0521	HOMO → LUMO+3 (9.0%)
		HOMO−5 → LUMO+2 (10.9%)			HOMO → LUMO+4 (8.4%)
		HOMO−4 → LUMO+3 (7.7%)			HOMO → LUMO+5 (76.9%)
173.4	0.0315	HOMO−7 → LUMO+1 (12.8%)	249.6	0.3958	HOMO → LUMO+1 (10.6%)
		HOMO−6 → LUMO+1 (16.9%)			HOMO → LUMO+2 (8.3%)
		HOMO−5 → LUMO+1 (8.3%)			HOMO → LUMO+3 (55.2%)
		HOMO−4 → LUMO+3 (30.5%)			HOMO → LUMO+5 (10.4%)
			275.5	0.0308	HOMO → LUMO+2 (61.6%)
					HOMO → LUMO+3 (23.7%)

**Table 7:** Signals for the calculated ^1^H NMR spectra of the ground states of the optimized structures of the keto-oxime, keto-hydroxylamine, and hydroxyl-oxime tautomers of molnupiravir, obtained by using the DFT/GIAO/B3LYP/6-311++G(2d,p) method (see Figure 3 for atoms labelling).

Hydrogen	Keto-oxime	Keto-hydroxylamine	Hydroxyl-oxime
H1	2.74	2.90	2.33
H2	5.47	6.24	2.87
H3	7.54	6.35	4.89
H4	5.29	7.88	6.82
H5	4.59	4.61	4.54
H6	5.39	5.55	5.59
H7	3.82	3.77	3.82
H8	4.40	4.38	4.35
H9	7.07	7.80	6.51
H10	5.39	5.24	5.84
H11	3.54	3.53	3.59
H12	3.96	3.95	4.05
H13	2.41	2.38	2.46
H14	0.96–1.06	1.02–1.06	1.03–1.06
H15	0.96–1.06	0.94–0.95	1.03–1.06
H16	0.96–1.06	0.94–0.95	0.96
H17	1.59	1.60	1.62
H18	0.73	0.71	0.76
H19	0.96–1.06	1.02–1.06	1.03–1.06

**Table 8:** Nonlinear optical parameters for the ground state of the optimized structure of the keto-oxime, keto-hydroxylamine and hydroxyl-oxime tautomers of molnupiravir, and urea [29], obtained by using the DFT/B3LYP/6-311++G(d,p) method ^1^.

Parameter	Keto-oxime	Keto-hydroxylamine	Hydroxyl-oxime	Urea [29]
μ_x_ (Debye)	−2.7182	−0.6997	1.0585	
μ_y_ (Debye)	−4.9139	−7.5858	1.0487	
μ_z_ (Debye)	−0.0544	−1.3843	0.7004	
μ_D_ (Debye)	5.6159	7.7428	1.6465	
α_xx_ (a.u.)	256.320	254.188	272.451	
α_yy_ (a.u.)	213.808	216.526	212.955	
α_zz_ (a.u.)	153.182	152.609	144.264	
α_xy_ (a.u.)	26.209	22.665	22.940	
α_xz_ (a.u.)	−14.113	−12.219	−12.450	
α_yz_ (a.u.)	6.759	9.801	1.162	
α (a.u.)	207.770	207.774	208.89	
α (esu)	30.792 × 10^−24^	30.792 × 10^−24^	31.106 × 10^−24^	3.8312 × 10^−24^
α_tautomer_/α_urea_	8.0	8.0	8.1	
Δα (a.u.)	104.189	100.937	119.970	
Δα (esu)	15.441 × 10^−24^	14.959 × 10^−24^	17.780 × 10^−24^	
β_xxx_ (a.u.)	−263.723	−47.212	16.843	
β_yyy_ (a.u.)	−5.671	−49.597	46.796	
β_zzz_ (a.u.)	2.192	0.908	0.007	
β_xyy_ (a.u.)	−0.326	−25.778	53.700	
β_xxy_ (a.u.)	−86.296	−66.014	130.374	
β_xxz_ (a.u.)	23.622	−18.584	6.807	
β_xzz_ (a.u.)	9.757	13.287	11.574	
β_yzz_ (a.u.)	−4.432	−2.785	5.898	
β_yyz_ (a.u.)	5.210	−7.786	4.231	
β_xyz_ (a.u.)	18.340	−1.679	−11.407	
β (a.u.)	273.715	135.020	200.946	
β (esu)	2.365 × 10^−30^	1.166 × 10^−30^	1.736 × 10^−30^	0.1947 × 10^−30^
β_tautomer_/β_urea_	12.1	6.0	8.9	

^1^ For α 1 a.u. = 0.1482 × 10^−24^ esu, for β 1 a.u. = 8.6393 × 10^−33^ esu.

**Table 9:** The best poses of the keto-oxime, keto-hydroxylamine, and hydroxyl-oxime tautomers of molnupiravir inside the binding sites of the listed proteins.

Protein	PDB Code	Keto-oxime	Keto-hydroxylamine	Hydroxyl-oxime
Main protease (Mpro)	6LU7	−6.60	−7.00	−7.30
Papain-like protease (PLpro)	6WUU	−7.50	−7.40	−7.40
Nonstructural protein 3 (Nsp3_range 207–379-AMP)	6W6Y	−6.90	−7.20	−6.90
Nonstructural protein 3 (Nsp3_range 207–379-MES)	6W6Y	−8.10	**−7.90**	−7.80
Helicase (Nsp13)-adp	6JYT	−6.30	−6.50	−6.20
Helicase (Nsp13)-ncb	6JYT	−6.80	−6.90	−6.60
RdRp-RTP	7BV2	**−9.90**	−7.50	**−9.30**
RdRp-RNA	7BV2	−7.00	−6.60	−6.80
Nsp14 (ExoN)	5C8S	−6.80	−7.00	−6.60
Nsp14 (N7-MTase)	5C8S	−7.50	−7.80	−7.80
Nsp15 (endoribonuclease)	6WLC	−6.30	−6.40	−6.50
Nsp16 (GTA site)	6WVN	−7.70	−7.70	−7.60
Nsp16 (MGP site)	6WVN	−6.30	−6.10	−6.10
Nsp16 (SAM site)	6WVN	−7.60	−7.30	−7.40
N protein (NCB site)	6WXD	−6.90	−7.20	−6.80
Spike protein, RBD (Native)	6M0J	−5.75	−5.91	−5.18
Spike protein, RBD (Mutated)	6M0J	−5.88	−5.85	−5.74

## Data Availability

All the data supporting the conclusions is included within the manuscript and is available on request from the corresponding authors.

## Supplementary Materials

The following supporting information can be downloaded at: https://www.mdpi.com/1422-0067/23/3/1508/s1.
