# Correction: Sharov et al. Computational Analysis of Molnupiravir. *Int. J. Mol. Sci.* 2022, *23*, 1508

**DOI:** 10.3390/ijms232113026

**Published:** 2022-10-27

**Authors:** Artem V. Sharov, Tatyana M. Burkhanova, Tugba Taskın Tok, Maria G. Babashkina, Damir A. Safin

**Affiliations:** 1 «Advanced Materials for Industry and Biomedicine» Laboratory, Kurgan State University, Sovetskaya Str. 63/4, 640020 Kurgan, Russia; 2 Center for Enterprise Relations, Ural Federal University Named after the First President of Russia B.N. Yeltsin, Mira Str. 19, 620002 Ekaterinburg, Russia; 3 Innovation Center for Chemical and Pharmaceutical Technologies, Ural Federal University Named after the First President of Russia B.N. Yeltsin, Mira Str. 19, 620002 Ekaterinburg, Russia; 4 Institute of Chemistry, University of Tyumen, Volodarskogo Str. 6, 625003 Tyumen, Russia; 5 Department of Chemistry, Faculty of Arts and Sciences, University of Gaziantep, Gaziantep 27310, Turkey; 6 Department of Bioinformatics and Computational Biology, Institute of Health Sciences, University of Gaziantep, Gaziantep 27310, Turkey

## Affiliation Correction

We received a complaint from the Université Catholique de Louvain. In the published version of the article [1], there was an error regarding the affiliation for Maria G. Babashkina. The correct affiliation is ^1^«Advanced Materials for Industry and Biomedicine» Laboratory, Kurgan State University, Sovetskaya Str. 63/4, 640020 Kurgan, Russia. The authors apologize for any inconvenience caused and state that the scientific conclusions are unaffected. The authors agree with this correction. This correction was approved by the Academic Editor. The original publication has also been updated.
