# Supplementary material for: Computational Analysis of Molnupiravir
Source: Int J Mol Sci. 2022 Jan 28;23(3):1508. doi: 10.3390/ijms23031508 (PMC8835990; doi:10.3390/ijms23031508)
Supplement: Supplementary file 1 [file ijms-23-01508-s001.zip › ijms-1554901-supplementary - final/ijms-1554901-supplementary - final.pdf]

Supplementary Materials

Computational Analysis of Molnupiravir

Artem V. Sharov [1,2], Tatyana M. Burkhanova [1,2,3], Tugba Taskın Tok [4,5,*], Maria G. Babashkina [6] and Damir A. Safin [1,2,3,*]

[1] «Advanced Materials for Industry and Biomedicine» Laboratory, Kurgan State University, Sovetskaya Str.63/4, 640020 Kurgan, Russia; sharow84@gmail.com (A.V.S.); t.m.burkhanova@utmn.ru (T.M.B.)
[2] Center for Enterprise Relations, Ural Federal University Named after the First President of Russia B.N. Yeltsin, Mira Str. 19, 620002 Ekaterinburg, Russia
[3] Innovation Center for Chemical and Pharmaceutical Technologies, Ural Federal University Named after the First President of Russia B.N. Yeltsin, Mira Str. 19, 620002 Ekaterinburg, Russia
[4] University of Tyumen, Volodarskogo Str. 6, 625003 Tyumen, Russia
[5] Department of Chemistry, Faculty of Arts and Sciences, University of Gaziantep, Gaziantep 27310, Turkey; ttaskin@gantep.edu.tr or taskin.tugba@gmail.com
6 Department of Bioinformatics and Computational Biology, Institute of Health Sciences, University of Gaziantep, Gaziantep 27310, Turkey
[7] Institute of Condensed Matter and Nanosciences, Université Catholique de Louvain, Place L. Pasteur 1, 1348 Louvain-la-Neuve, Belgium; maria.babashkina@mail.ru
* Correspondence: damir.a.safin@gmail.com

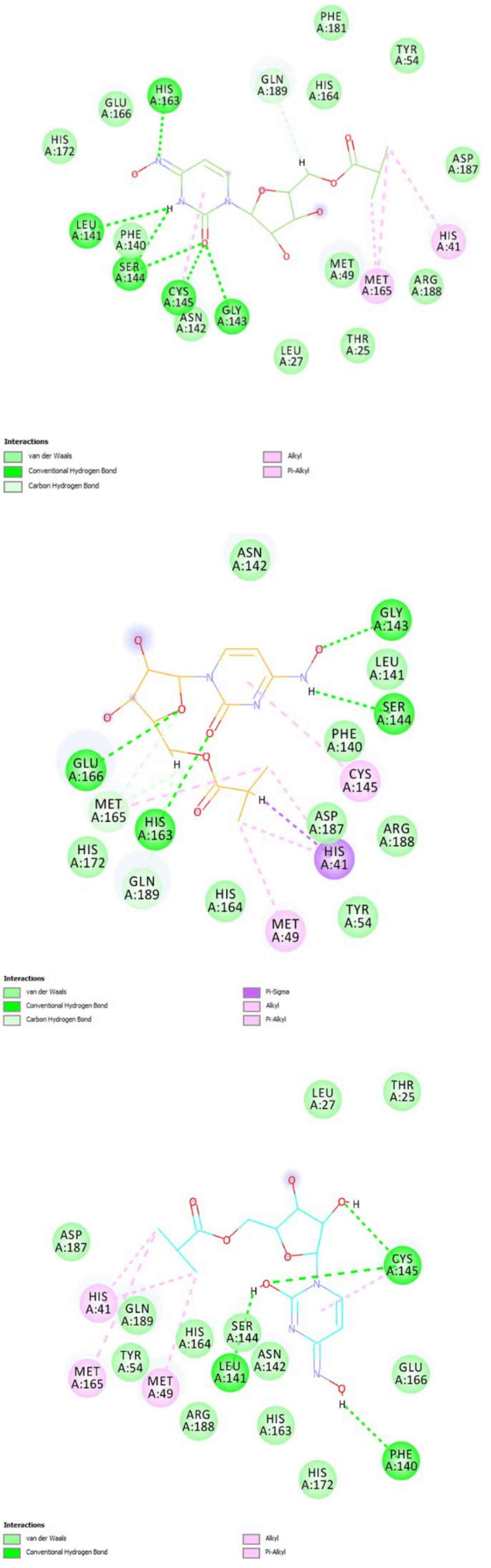

Figure S1. 2D views on the interaction of the keto-oxime (top), keto-hydroxylamine (middle) and hydroxyl-oxime (bottom) tautomers of molnupiravir with Mpro.

Table S1. Best types of interactions and distances of the keto-oxime, keto-hydroxylamine and hydroxyl-oxime tautomers of molnupiravir with RdRp-RTP and Nonstructural protein 3 (Nsp3_range 207–379-MES).

**Interactions: RdRp-RTP–keto-oxime**	**Distance Å**	**Bonding**	**Bonding Types**	**Binding site of enzyme**	**Binding site of ligand**
A:LYS593:HN - d:Keto-oxime:O	2.2011	Hydrogen Bond	Conventional Hydrogen Bond	A:LYS593:HN	d:Keto-oxime:O
T:A13:H3 - d:Keto-oxime:O	2.8657	Hydrogen Bond	Conventional Hydrogen Bond	T:A13:H3	d:Keto-oxime:O
T:A14:HO2' - d:Keto-oxime:O	2.5678	Hydrogen Bond	Conventional Hydrogen Bond	T:A14:HO2'	d:Keto-oxime:O
d:Keto-oxime:H - P:U18:O2'	2.6897	Hydrogen Bond	Conventional Hydrogen Bond	P:U18:O2'	d:Keto-oxime:H
d:Keto-oxime:H - A:THR591:O	1.7369	Hydrogen Bond	Conventional Hydrogen Bond	A:THR591:O	d:Keto-oxime:H
d:Keto-oxime:H - A:THR591:O	1.9995	Hydrogen Bond	Conventional Hydrogen Bond	A:THR591:O	d:Keto-oxime:H
A:SER592:HA - d:Keto-oxime:O	2.5107	Hydrogen Bond	Carbon Hydrogen Bond	A:SER592:HA	d:Keto-oxime:O
P:U18:H1' - d:Keto-oxime:O	2.4402	Hydrogen Bond	Carbon Hydrogen Bond	P:U18:H1'	d:Keto-oxime:O
T:A14:H4' - d:Keto-oxime:O	3.0799	Hydrogen Bond	Carbon Hydrogen Bond	T:A14:H4'	d:Keto-oxime:O
T:A14:H1' - d:Keto-oxime:O	3.0285	Hydrogen Bond	Carbon Hydrogen Bond	T:A14:H1'	d:Keto-oxime:O
d:Keto-oxime:H12 - P:A19:O2'	2.6305	Hydrogen Bond	Carbon Hydrogen Bond	P:A19:O2'	d:Keto-oxime:H12
A:LYS593:NZ - d:Keto-oxime	4.2440	Electrostatic	Pi-Cation	A:LYS593:NZ	d:Keto-oxime
T:A14 - d:Keto-oxime	5.5050	Hydrophobic	Pi-Pi T-shaped	T:A14	d:Keto-oxime
A:ALA688 - d:Keto-oxime:C	3.9193	Hydrophobic	Alkyl	A:ALA688	d:Keto-oxime:C
d:Keto-oxime:C - A:ILE589	3.9070	Hydrophobic	Alkyl	A:ILE589	d:Keto-oxime:C
d:Keto-oxime:C - A:LEU758	4.9149	Hydrophobic	Alkyl	A:LEU758	d:Keto-oxime:C
d:Keto-oxime:C - A:ILE589	3.7806	Hydrophobic	Alkyl	A:ILE589	d:Keto-oxime:C
d:Keto-oxime:C - A:LEU758	4.5327	Hydrophobic	Alkyl	A:LEU758	d:Keto-oxime:C
d:Keto-oxime - A:LYS593	4.4077	Hydrophobic	Pi-Alkyl	A:LYS593	d:Keto-oxime
**Interactions: Nsp3_range 207–379-MES–keto-hydroxylamine**	**Distance Å**	**Bonding**	**Bonding Types**	**Binding site of enzyme**	**Binding site of ligand**
B:ASN40:HD22 - d:Keto-hydroxylamine:O	2.0675	Hydrogen Bond	Conventional Hydrogen Bond	B:ASN40:HD22	d:Keto-hydroxylamine:O
B:GLY46:HN - d:Keto-hydroxylamine:O	2.1536	Hydrogen Bond	Conventional Hydrogen Bond	B:GLY46:HN	d:Keto-hydroxylamine:O
B:VAL49:HN - d:Keto-hydroxylamine:O	2.2900	Hydrogen Bond	Conventional Hydrogen Bond	B:VAL49:HN	d:Keto-hydroxylamine:O
B:ALA50:HN - d:Keto-hydroxylamine:O	2.2981	Hydrogen Bond	Conventional Hydrogen Bond	B:ALA50:HN	d:Keto-hydroxylamine:O
d:Keto-hydroxylamine:H - B:ALA38:O	1.8839	Hydrogen Bond	Conventional Hydrogen Bond	B:ALA38:O	d:Keto-hydroxylamine:H
d:Keto-hydroxylamine:H6 - B:GLY47:O	2.6752	Hydrogen Bond	Carbon Hydrogen Bond	B:GLY47:O	d:Keto-hydroxylamine:H6
B:PHE132 - d:Keto-hydroxylamine	4.8548	Hydrophobic	Pi-Pi T-shaped	B:PHE132	d:Keto-hydroxylamine
B:PHE132 - d:Keto-hydroxylamine:C	4.9872	Hydrophobic	Pi-Alkyl	B:PHE132	d:Keto-hydroxylamine:C
d:Keto-hydroxylamine - B:ALA38	4.0945	Hydrophobic	Pi-Alkyl	B:ALA38	d:Keto-hydroxylamine

d:Keto-hydroxylamine - B:ILE131	5.4535	Hydrophobic	Pi-Alkyl	B:ILE131	d:Keto-hydroxylamine
**Interactions: RdRp-RTP–hydroxyl-oxime**	**Distance Å**	**Bonding**	**Bonding Types**	**Binding site of enzyme**	**Binding site of ligand**
A:LYS593:HN - d:Hydroxyl-oxime:O	2.5103	Hydrogen Bond	Conventional Hydrogen Bond	A:LYS593:HN	d:Hydroxyl-oxime:O
P:A19:H3 - d:Hydroxyl-oxime:O	2.0424	Hydrogen Bond	Conventional Hydrogen Bond	P:A19:H3	d:Hydroxyl-oxime:O
T:A13:H3 - d:Hydroxyl-oxime:O	2.5232	Hydrogen Bond	Conventional Hydrogen Bond	T:A13:H3	d:Hydroxyl-oxime:O
d:Hydroxyl-oxime:H - A:THR591:O	2.4919	Hydrogen Bond	Conventional Hydrogen Bond	A:THR591:O	d:Hydroxyl-oxime:H
d:Hydroxyl-oxime:H - A:THR591:O	3.0054	Hydrogen Bond	Conventional Hydrogen Bond	A:THR591:O	d:Hydroxyl-oxime:H
A:LYS593:NZ - d:Hydroxyl-oxime	3.5954	Electrostatic	Pi-Cation	A:LYS593:NZ	d:Hydroxyl-oxime
A:ALA688 - d:Hydroxyl-oxime:C	4.4898	Hydrophobic	Alkyl	A:ALA688	d:Hydroxyl-oxime:C
d:Hydroxyl-oxime:C - A:ILE589	4.1574	Hydrophobic	Alkyl	A:ILE589	d:Hydroxyl-oxime:C
d:Hydroxyl-oxime:C - A:LEU758	4.6112	Hydrophobic	Alkyl	A:LEU758	d:Hydroxyl-oxime:C
d:Hydroxyl-oxime:C - A:ILE589	3.9944	Hydrophobic	Alkyl	A:ILE589	d:Hydroxyl-oxime:C
d:Hydroxyl-oxime:C - A:LEU758	4.3221	Hydrophobic	Alkyl	A:LEU758	d:Hydroxyl-oxime:C
d:Hydroxyl-oxime - A:LYS593	4.4437	Hydrophobic	Pi-Alkyl	A:LYS593	d:Hydroxyl-oxime

Table S2. Best types of interactions and distances of the keto-oxime, keto-hydroxylamine and hydroxyl-oxime tautomers of molnupiravir with Mpro.

Interactions: Mpro–keto-oxime	Distance Å	Bonding	Bonding Types	Binding site of enzyme	Binding site of ligand
A:GLY143:HN - d:Keto-oxime:O	2.0634	Hydrogen Bond	Conventional Hydrogen Bond	A:GLY143:HN	d:Keto-oxime:O
A:SER144:HN - d:Keto-oxime:O	2.5457	Hydrogen Bond	Conventional Hydrogen Bond	A:SER144:HN	d:Keto-oxime:O
A:CYS145:HN - d:Keto-oxime:O	2.7296	Hydrogen Bond	Conventional Hydrogen Bond	A:CYS145:HN	d:Keto-oxime:O
A:HIS163:HE2 - d:Keto-oxime:N	1.9378	Hydrogen Bond	Conventional Hydrogen Bond	A:HIS163:HE2	d:Keto-oxime:N
d:Keto-oxime:H - A:LEU141:O	2.3031	Hydrogen Bond	Conventional Hydrogen Bond	A:LEU141:O	d:Keto-oxime:H
d:Keto-oxime:H - A:SER144:OG	2.3839	Hydrogen Bond	Conventional Hydrogen Bond	A:SER144:OG	d:Keto-oxime:H
d:Keto-oxime:H12 - A:GLN189:OE1	2.4764	Hydrogen Bond	Carbon Hydrogen Bond	A:GLN189:OE1	d:Keto-oxime:H12
d:Keto-oxime:C - A:MET165	4.6008	Hydrophobic	Alkyl	A:MET165	d:Keto-oxime:C
d:Keto-oxime:C - A:MET165	4.3516	Hydrophobic	Alkyl	A:MET165	d:Keto-oxime:C
A:HIS41 - d:Keto-oxime:C	3.7850	Hydrophobic	Pi-Alkyl	A:HIS41	d:Keto-oxime:C
d:Keto-oxime - A:CYS145	4.7903	Hydrophobic	Pi-Alkyl	A:CYS145	d:Keto-oxime
**Interactions: Mpro–keto-hydroxylamine**	**Distance Å**	**Bonding**	**Bonding Types**	**Binding site of enzyme**	**Binding site of ligand**
A:GLY143:HN - d:Keto-hydroxylamine:O	2.0907	Hydrogen Bond	Conventional Hydrogen Bond	A:GLY143:HN	d:Keto-hydroxylamine:O
A:HIS163:HE2 - d:Keto-hydroxylamine:O	2.2237	Hydrogen Bond	Conventional Hydrogen Bond	A:HIS163:HE2	d:Keto-hydroxylamine:O
A:GLU166:HN - d:Keto-hydroxylamine:O	1.8749	Hydrogen Bond	Conventional Hydrogen Bond	A:GLU166:HN	d:Keto-hydroxylamine:O
d:Keto-hydroxylamine:H - A:LEU141:O	2.0278	Hydrogen Bond	Conventional Hydrogen Bond	A:LEU141:O	d:Keto-hydroxylamine:H
d:Keto-hydroxylamine:H - A:SER144:OG	2.8609	Hydrogen Bond	Conventional Hydrogen Bond	A:SER144:OG	d:Keto-hydroxylamine:H
d:Keto-hydroxylamine:H - A:GLU166:O	2.1302	Hydrogen Bond	Conventional Hydrogen Bond	A:GLU166:O	d:Keto-hydroxylamine:H
A:MET165:HA - d:Keto-hydroxylamine:O	2.4746	Hydrogen Bond	Carbon Hydrogen Bond	A:MET165:HA	d:Keto-hydroxylamine:O
d:Keto-hydroxylamine:H5 - A:GLU166:O	2.9808	Hydrogen Bond	Carbon Hydrogen Bond	A:GLU166:O	d:Keto-hydroxylamine:H5
d:Keto-hydroxylamine:H11 - A:GLN189:OE1	2.7586	Hydrogen Bond	Carbon Hydrogen Bond	A:GLN189:OE1	d:Keto-hydroxylamine:H11
d:Keto-hydroxylamine:H12 - A:GLN189:OE1	2.3338	Hydrogen Bond	Carbon Hydrogen Bond	A:GLN189:OE1	d:Keto-hydroxylamine:H12
d:Keto-hydroxylamine:C - A:MET165	4.4796	Hydrophobic	Alkyl	A:MET165	d:Keto-hydroxylamine:C
d:Keto-hydroxylamine:C - A:MET49	3.6485	Hydrophobic	Alkyl	A:MET49	d:Keto-hydroxylamine:C
A:HIS41 - d:Keto-hydroxylamin:C	4.0395	Hydrophobic	Pi-Alkyl	A:HIS41	d:Keto-hydroxylamine:C
A:HIS41 - d:Keto-hydroxylamine:C	4.2457	Hydrophobic	Pi-Alkyl	A:HIS41	d:Keto-hydroxylamine:C
d:Keto-hydroxylamine - A:CYS145	4.9167	Hydrophobic	Pi-Alkyl	A:CYS145	d:Keto-hydroxylamine
**Interactions: Mpro–hydroxyl-oxime**	**Distance Å**	**Bonding**	**Bonding Types**	**Binding site of enzyme**	**Binding site of ligand**
A:CYS145:HN - d:Hydroxyl-oxime:O	2.6535	Hydrogen Bond	Conventional Hydrogen Bond	A:CYS145:HN	d:Hydroxyl-oxime:O

A:CYS145:HN - d:Hydroxyl-oxime:O	2.7224	Hydrogen Bond	Conventional Hydrogen Bond	A:CYS145:HN	d:Hydroxyl-oxime:O
d:Hydroxyl-oxime:H - A:LEU141:O	2.3179	Hydrogen Bond	Conventional Hydrogen Bond	A:LEU141:O	d:Hydroxyl-oxime:H
d:Hydroxyl-oxime:H - A:PHE140:O	2.4855	Hydrogen Bond	Conventional Hydrogen Bond	A:PHE140:O	d:Hydroxyl-oxime:H
d:Hydroxyl-oxime:C - A:MET165	4.8450	Hydrophobic	Alkyl	A:MET165	d:Hydroxyl-oxime:C
d:Hydroxyl-oxime:C - A:MET49	3.5653	Hydrophobic	Alkyl	A:MET49	d:Hydroxyl-oxime:C
A:HIS41 - d:Hydroxyl-oxime:C	3.6874	Hydrophobic	Pi-Alkyl	A:HIS41	d:Hydroxyl-oxime:C
A:HIS41 - d:Hydroxyl-oxime:C	4.5811	Hydrophobic	Pi-Alkyl	A:HIS41	d:Hydroxyl-oxime:C
d:Hydroxyl-oxime - A:CYS145	4.5139	Hydrophobic	Pi-Alkyl	A:CYS145	d:Hydroxyl-oxime

Table S3. Best types of interactions and distances of the keto-oxime, keto-hydroxylamine and hydroxyl-oxime tautomers of molnupiravir with native and mutated Spike protein, RDB.

Interactions: native Spike protein, RDB–keto-oxime	Distance Å	Bonding	Bonding Types	Binding site of enzyme	Binding site of ligand
E:THR430:HG1 - :Keto-oxime:O	1.7665	Hydrogen Bond	Conventional Hydrogen Bond	E:THR430:HG1	:Keto-oxime:O
E:PHE515:HN - :Keto-oxime:O	2.3622	Hydrogen Bond	Conventional Hydrogen Bond	E:PHE515:HN	:Keto-oxime:O
:Keto-oxime:H - E:PHE515:O	2.6361	Hydrogen Bond	Conventional Hydrogen Bond	E:PHE515:O	:Keto-oxime:H
:Keto-oxime:H - E:GLU516:OE1	1.9866	Hydrogen Bond	Conventional Hydrogen Bond	E:GLU516:OE1	:Keto-oxime:H
:Keto-oxime:H - E:GLU516:OE1	1.9311	Hydrogen Bond	Conventional Hydrogen Bond	E:GLU516:OE1	:Keto-oxime:H
:Keto-oxime:H6 - E:GLU516:OE1	2.8925	Hydrogen Bond	Carbon Hydrogen Bond	E:GLU516:OE1	:Keto-oxime:H6
:Keto-oxime:C - E:PRO463	4.6505	Hydrophobic	Alkyl	E:PRO463	:Keto-oxime:C
E:PHE464 - :Keto-oxime:C	4.8290	Hydrophobic	Pi-Alkyl	E:PHE464	:Keto-oxime:C
E:PHE464 - :Keto-oxime:C	5.3161	Hydrophobic	Pi-Alkyl	E:PHE464	:Keto-oxime:C
**Interactions: native Spike protein, RDB–keto-hydroxylamine**	**Distance Å**	**Bonding**	**Bonding Types**	**Binding site of enzyme**	**Binding site of ligand**
E:SER399:HG - :Keto- Hydroxylamine:O	3.0715	Hydrogen Bond	Conventional Hydrogen Bond	E:SER399:HG	:Keto- Hydroxylamine:O
:Keto- Hydroxylamine:H - E:GLU340:OE2	2.0541	Hydrogen Bond	Conventional Hydrogen Bond	E:GLU340:OE2	:Keto- Hydroxylamine:H
:Keto- Hydroxylamine:H - E:SER399:OG	2.4085	Hydrogen Bond	Conventional Hydrogen Bond	E:SER399:OG	:Keto- Hydroxylamine:H
:Keto- Hydroxylamine:H - E:SER399:OG	1.9557	Hydrogen Bond	Conventional Hydrogen Bond	E:SER399:OG	:Keto- Hydroxylamine:H
:Keto- Hydroxylamine:H7 - E:ARG346:O	2.8351	Hydrogen Bond	Carbon Hydrogen Bond	E:ARG346:O	:Keto- Hydroxylamine:H7
:Keto- Hydroxylamine:H11 - E:ARG346:O	2.9918	Hydrogen Bond	Carbon Hydrogen Bond	E:ARG346:O	:Keto- Hydroxylamine:H11
E:ASN354:ND2 - :Keto- Hydroxylamine	4.1573	Hydrogen Bond	Pi-Donor Hydrogen Bond	E:ASN354:ND2	:Keto- Hydroxylamine
:Keto- Hydroxylamine:C - E:ARG346	4.3579	Hydrophobic	Alkyl	E:ARG346	:Keto- Hydroxylamine:C
:Keto- Hydroxylamine - E:VAL341	4.9488	Hydrophobic	Pi-Alkyl	E:VAL341	:Keto- Hydroxylamine
:Keto- Hydroxylamine - E:LYS356	4.7270	Hydrophobic	Pi-Alkyl	E:LYS356	:Keto- Hydroxylamine
**Interactions: native Spike protein, RDB–hydroxyl-oxime**	**Distance Å**	**Bonding**	**Bonding Types**	**Binding site of enzyme**	**Binding site of ligand**
:Hydroxyl-oxime:H - E:GLU484:OE1	1.7532	Hydrogen Bond	Conventional Hydrogen Bond	E:GLU484:OE1	:Hydroxyl-oxime:H
:Hydroxyl-oxime:H - E:GLU484:OE2	2.1540	Hydrogen Bond	Conventional Hydrogen Bond	E:GLU484:OE2	:Hydroxyl-oxime:H
:Hydroxyl-oxime:H - E:GLU484:OE2	1.9856	Hydrogen Bond	Conventional Hydrogen Bond	E:GLU484:OE2	:Hydroxyl-oxime:H
E:SER494:HB1 - :Hydroxyl-oxime:O	2.4070	Hydrogen Bond	Carbon Hydrogen Bond	E:SER494:HB1	:Hydroxyl-oxime:O
:Hydroxyl-oxime:H6 - E:GLU484:OE1	2.2942	Hydrogen Bond	Carbon Hydrogen Bond	E:GLU484:OE1	:Hydroxyl-oxime:H6
:Hydroxyl-oxime:H10 - E:PHE490:O	2.6683	Hydrogen Bond	Carbon Hydrogen Bond	E:PHE490:O	:Hydroxyl-oxime:H10
:Hydroxyl-oxime:H10 - E:LEU492:O	2.3591	Hydrogen Bond	Carbon Hydrogen Bond	E:LEU492:O	:Hydroxyl-oxime:H10

:Hydroxyl-oxime:H12 - E:LEU492:O	2.7154	Hydrogen Bond	Carbon Hydrogen Bond	E:LEU492:O	:Hydroxyl-oxime:H12
:Hydroxyl-oxime:C - E:LEU452	3.9875	Hydrophobic	Alkyl	E:LEU452	:Hydroxyl-oxime:C
:Hydroxyl-oxime:C - E:LEU452	5.2117	Hydrophobic	Alkyl	E:LEU452	:Hydroxyl-oxime:C
**Interactions: mutated Spike protein, RDB–keto-oxime**	**Distance Å**	**Bonding**	**Bonding Types**	**Binding site of enzyme**	**Binding site of ligand**
E:GLY431:HN - :Keto-oxime:O	2.0845	Hydrogen Bond	Conventional Hydrogen Bond	E:GLY431:HN	:Keto-oxime:O
:Keto-oxime:H - E:PHE515:O	2.5983	Hydrogen Bond	Conventional Hydrogen Bond	E:PHE515:O	:Keto-oxime:H
:Keto-oxime:H - E:PHE515:O	3.0184	Hydrogen Bond	Conventional Hydrogen Bond	E:PHE515:O	:Keto-oxime:H
:Keto-oxime:H - E:GLU516:OE1	1.9932	Hydrogen Bond	Conventional Hydrogen Bond	E:GLU516:OE1	:Keto-oxime:H
:Keto-oxime:H - E:GLU516:OE1	2.0186	Hydrogen Bond	Conventional Hydrogen Bond	E:GLU516:OE1	:Keto-oxime:H
E:SER514:HA - :Keto-oxime:O	2.8766	Hydrogen Bond	Carbon Hydrogen Bond	E:SER514:HA	:Keto-oxime:O
:Keto-oxime:H6 - E:GLU516:OE1	2.8750	Hydrogen Bond	Carbon Hydrogen Bond	E:GLU516:OE1	:Keto-oxime:H6
:Keto-oxime:C - E:PRO426	4.6574	Hydrophobic	Alkyl	E:PRO426	:Keto-oxime:C
:Keto-oxime:C - E:PRO463	4.4017	Hydrophobic	Alkyl	E:PRO463	:Keto-oxime:C
E:PHE464 - :Keto-oxime:C	4.7589	Hydrophobic	Pi-Alkyl	E:PHE464	:Keto-oxime:C
E:PHE464 - :Keto-oxime:C	5.0872	Hydrophobic	Pi-Alkyl	E:PHE464	:Keto-oxime:C
**Interactions: mutated Spike protein, RDB–keto-hydroxylamine**	**Distance Å**	**Bonding**	**Bonding Types**	**Binding site of enzyme**	**Binding site of ligand**
E:ALA348:HN - :Keto-Hydroxylamine:O	2.1055	Hydrogen Bond	Conventional Hydrogen Bond	E:ALA348:HN	:Keto-Hydroxylamine:O
E:SER399:HG - :Keto-Hydroxylamine:O	3.0495	Hydrogen Bond	Conventional Hydrogen Bond	E:SER399:HG	:Keto-Hydroxylamine:O
:Keto-Hydroxylamine:H - E:GLU340:OE2	2.1062	Hydrogen Bond	Conventional Hydrogen Bond	E:GLU340:OE2	:Keto-Hydroxylamine:H
:Keto-Hydroxylamine:H - E:GLU340:OE2	2.1042	Hydrogen Bond	Conventional Hydrogen Bond	E:GLU340:OE2	:Keto-Hydroxylamine:H
:Keto-Hydroxylamine:H - E:SER399:OG	1.9616	Hydrogen Bond	Conventional Hydrogen Bond	E:SER399:OG	:Keto-Hydroxylamine:H
:Keto-Hydroxylamine:H - E:SER399:OG	1.7617	Hydrogen Bond	Conventional Hydrogen Bond	E:SER399:OG	:Keto-Hydroxylamine:H
:Keto-Hydroxylamine:H8 - E:ARG346:O	2.2937	Hydrogen Bond	Carbon Hydrogen Bond	E:ARG346:O	:Keto-Hydroxylamine:H8
:Keto-Hydroxylamine:H11 - E:ARG346:O	1.8321	Hydrogen Bond	Carbon Hydrogen Bond	E:ARG346:O	:Keto-Hydroxylamine:H11
E:ALA348 - :Keto-Hydroxylamine:C	3.9664	Hydrophobic	Alkyl	E:ALA348	:Keto-Hydroxylamine:C
E:ALA348 - :Keto-Hydroxylamine:C	3.4386	Hydrophobic	Alkyl	E:ALA348	:Keto-Hydroxylamine:C
:Keto-Hydroxylamine - E:VAL341	4.3749	Hydrophobic	Pi-Alkyl	E:VAL341	:Keto-Hydroxylamine
:Keto-Hydroxylamine - E:ALA344	5.1236	Hydrophobic	Pi-Alkyl	E:ALA344	:Keto-Hydroxylamine
:Keto-Hydroxylamine - E:LYS356	4.5973	Hydrophobic	Pi-Alkyl	E:LYS356	:Keto-Hydroxylamine
**Interactions: mutated Spike protein, RDB–hydroxyl-oxime**	**Distance Å**	**Bonding**	**Bonding Types**	**Binding site of enzyme**	**Binding site of ligand**
:Hydroxyl-oxime:H - E:GLU484:OE1	1.8041	Hydrogen Bond	Conventional Hydrogen Bond	E:GLU484:OE1	:Hydroxyl-oxime:H

:Hydroxyl-oxime:H - E:GLU484:OE1	1.8056	Hydrogen Bond	Conventional Hydrogen Bond	E:GLU484:OE1	:Hydroxyl-oxime:H
:Hydroxyl-oxime:H - E:GLU484:OE2	2.1801	Hydrogen Bond	Conventional Hydrogen Bond	E:GLU484:OE2	:Hydroxyl-oxime:H
E:GLN493:HA - :Hydroxyl-oxime:O	2.2138	Hydrogen Bond	Carbon Hydrogen Bond	E:GLN493:HA	:Hydroxyl-oxime:O
:Hydroxyl-oxime:H6 - E:GLU484:OE1	1.9583	Hydrogen Bond	Carbon Hydrogen Bond	E:GLU484:OE1	:Hydroxyl-oxime:H6
:Hydroxyl-oxime:H11 - E:GLN493:OE1	2.4637	Hydrogen Bond	Carbon Hydrogen Bond	E:GLN493:OE1	:Hydroxyl-oxime:H11
:Hydroxyl-oxime:H12 - E:LEU492:O	1.8804	Hydrogen Bond	Carbon Hydrogen Bond	E:LEU492:O	:Hydroxyl-oxime:H12
:Hydroxyl-oxime:C - E:LEU452	3.9903	Hydrophobic	Alkyl	E:LEU452	:Hydroxyl-oxime:C
:Hydroxyl-oxime:C - E:LEU492	5.3971	Hydrophobic	Alkyl	E:LEU492	:Hydroxyl-oxime:C
E:PHE490 - :Hydroxyl-oxime:C	4.4689	Hydrophobic	Pi-Alkyl	E:PHE490	:Hydroxyl-oxime:C
E:PHE490 - :Hydroxyl-oxime:C	4.6738	Hydrophobic	Pi-Alkyl	E:PHE490	:Hydroxyl-oxime:C
